# Recent Advances and Perspectives in Relation to the Metabolomics-Based Study of Diabetic Retinopathy

**DOI:** 10.3390/metabo13091007

**Published:** 2023-09-12

**Authors:** Shuling He, Lvyun Sun, Jiali Chen, Yang Ouyang

**Affiliations:** Department of Health Inspection and Quarantine, School of Public Health, Fujian Medical University, Fuzhou 350122, China; heshuling@fjmu.edu.cn (S.H.);

**Keywords:** diabetic retinopathy, metabolomics, biomarker, metabolic pathway

## Abstract

Diabetic retinopathy (DR), a prevalent microvascular complication of diabetes, is a major cause of acquired blindness in adults. Currently, a clinical diagnosis of DR primarily relies on fundus fluorescein angiography, with a limited availability of effective biomarkers. Metabolomics, a discipline dedicated to scrutinizing the response of various metabolites within living organisms, has shown noteworthy advancements in uncovering metabolic disorders and identifying key metabolites associated with DR in recent years. Consequently, this review aims to present the latest advancements in metabolomics techniques and comprehensively discuss the principal metabolic outcomes derived from analyzing blood, vitreous humor, aqueous humor, urine, and fecal samples.

## 1. Introduction

Diabetic retinopathy, as one of the various microvascular complications of diabetes, represents a significant contributor to adult acquired blindness [1,2]. Extensive analysis carried out by Yau et al. encompassing 35 studies comprising over 20,000 participants worldwide revealed that approximately 35% of individuals with diabetes exhibited some form of retinopathy [3]. Type 1 diabetes patients demonstrated an even higher incidence rate, with up to 54% of cases being affected [4]. Factors such as prolonged diabetes duration and an inadequate control of blood glucose and blood pressure emerge as the primary etiological elements underlying DR [3]. In light of these findings, a concerning projection indicates a surge in the number of DR cases from 103 million in 2020 to an estimated 161 million by 2045, signifying an escalating global burden [5]. The disease progression of DR can typically be categorized into two stages: non-proliferative DR (NPDR) and proliferative DR (PDR), based on the presence or absence of neovascularization within the retina. NPDR generally precedes the development of PDR [6]. This systematic classification provides valuable insights into the temporal sequence and severity of DR manifestations. Kornbla et al. conducted a comprehensive literature review on the adverse reactions associated with fluorescein angiography by utilizing the PubMed database. The analysis encompassed 78 relevant publications spanning from 1961 to 2017, revealing a diverse range of adverse reaction incidences, ranging from 0.083% to 21.69%. Among these cases, mild adverse reactions were reported to occur at rates of 1.24% to 17.65%, while moderate reactions were observed in 0.2% to 6% of instances. Severe adverse reactions were relatively infrequent, with reported occurrences ranging from 0.04% to 0.59% [7]. Deaths caused by fundus fluorescein angiography have also been reported [8,9]. The treatment options available for retinopathy management, such as laser photocoagulation, vitreoretinal surgery, and the intravitreal injection of corticosteroids or anti-vascular endothelial growth factor, are employed exclusively when the patient’s visual function is threatened. Nonetheless, these treatments are associated with potential side effects [10,11,12]. Although significant advancements have been made in retinal imaging technology in recent years, enabling enhanced retina structural visualization without the need for fluorescein administration [13], mild DR cases often exhibit subtle or no discernible abnormalities, leading to challenges in early diagnosis and subsequent intervention [14]. Hence, a comprehensive exploration of the metabolic characteristics of DR holds promise in elucidating its underlying pathogenesis, discovering potential key metabolites, and facilitating the development of novel clinical diagnostic approaches and treatment strategies.

Metabolomics, a systematic discipline, investigates the dynamic response of multiple metabolites within living organisms when subjected to internal genetic mutations, pathophysiological alterations, or external environmental stimuli [15]. It is a high-throughput analysis technology that can qualitatively or quantitatively study metabolites with a relative molecular weight less than 1500. Currently, metabolomics emerged as a pivotal tool extensively employed in diverse domains such as plant biology [16], nutrition [17], medicine [18,19], and clinical [20,21] research. Remarkable progress has been made by metabolomics in the discovery of metabolic disorders and key metabolites associated with DR. Thus, this paper provides a comprehensive summary of major improvements in analytical platforms and recent advances in metabolomics research, and discusses the advantages and limitations of each approach. Subsequently, we review the utility of metabolomics in DR studies, specifically focusing on the major metabolic outcomes observed in clinical populations through the analysis of blood, vitreous fluid, aqueous humor, urine, and stool samples. By exploring the metabolomics landscape, we aim to shed light on the metabolic intricacies underlying DR pathogenesis, paving the way for potential diagnostic and therapeutic avenues.

## 2. Analytical Technologies for Metabolomics

Analytical methods constitute the fundamental components of metabolomics research. Nuclear magnetic resonance spectroscopy (NMR) and mass spectrometry (MS) are the two predominant analytical techniques employed in metabolomics investigations [22]. Notably, chromatography-MS coupling systems, including gas chromatography-MS (GC-MS) and liquid chromatography-MS (LC-MS), have gained substantial popularity and represent the most frequently utilized methodologies. These coupling systems provide robust analytical capabilities, empowering researchers to comprehensively profile and characterize metabolites in various biological samples. Their wide-scale adoption in metabolomics research underscores their efficacy and versatility in exploring the metabolic intricacies associated with DR.

### 2.1. Nuclear Magnetic Resonance Spectroscopy

NMR is a widely utilized tool for metabolite identification due to its distinctive characteristics. It offers simplicity in sample preparation, no damage to the structure and properties of the sample, good reproducibility, short analysis time, robust signal detection, and the capability for absolute quantification of metabolites [23]. ^1^H-NMR is particularly extensively employed in metabolomics research as hydrogen atoms are prevalent in the majority of organic metabolites [24]. In the realm of biomolecular NMR, crucial nuclei like ^13^C, ^15^N, and ^31^P play pivotal roles. For instance, ^13^C NMR facilitates structure elucidation and molecular identification [25], while ^31^P NMR offers a broad chemical shift range and sharp peaks. Nonetheless, the overlapping signals of many phosphorylated compounds pose challenges for ^31^P NMR analysis [26]. It is worth noting that one-dimensional NMR techniques are relatively less sensitive, limiting their ability to detect metabolites present in low abundance. Two-dimensional NMR (2D NMR) has been developed to overcome the problem of overlapping resonance in one-dimensional NMR spectroscopy. It also provides enhanced sensitivity and the ability to detect and identify a broader range of metabolites. Within the field of metabolomics, techniques like heteronuclear single-quantum coherence spectroscopy (HSQC) and heteronuclear multiple-quantum correlation (HMQC) are predominantly employed. In addition, homonuclear experiments including correlated spectroscopy (COSY) [27] and total correlation spectroscopy (TOCSY) [28] play integral roles in metabolite characterization. Nonetheless, a significant drawback associated with 2D NMR techniques is the lengthy measurement time, which can extend to several hours for each sample, thus limiting their applicability to small sample sizes [29]. To address this limitation and expedite spectral acquisition without compromising sensitivity in high-throughput research, Ghosh et al. utilized the selective optimized flip-angle short-transient ^1^H-^13^C HMQC technique in combination with nonlinear sampling strategies. This approach allowed for the acquisition of urine and serum sample spectra with a significantly reduced experimental time, requiring only about one-seventh of the time compared to traditional ^1^H-^13^C HSQC experiments, while nearly retaining all molecular information [30]. Furthermore, recent years have witnessed remarkable advancements in NMR methods, such as hyperpolarized NMR [31] and cryogenic-probe-based Rheo-NMR [32], which have improved spectral resolution and metabolite identification ability. These developments hold promise for further enhancing the efficiency and accuracy of metabolomics studies.

### 2.2. Gas Chromatography-Mass Spectrometry

MS is a highly potent technique primarily employed for the identification of unknown compounds and the quantification of known molecules within samples. GC-MS possesses high sensitivity, good peak resolution, and extensive databases, rendering it suitable for the qualitative analysis of metabolites [33]. Leveraging the newly developed GC/quadrupole Orbitrap MS system for targeted metabolite analysis can enhance sensitivity and facilitate the utilization of quantitative strategies [34].

One inherent limitation of GC-MS is its restricted applicability to the separation and identification of low-molecular-weight (<650 Da) and volatile compounds. In order to detect polar, heat-resistant, non-volatile metabolites, chemical derivatization is necessitated prior to analysis [35]. The common metabolites typically analyzed using GC-MS analysis encompass amino acids, organic acids, and fatty acids [36], which predominantly pertain to biochemical processes such as the tricarboxylic acid (TCA) cycle, glycolysis, amino acid metabolism, and fatty acid metabolism. Cesare et al. have proposed an enhanced GC-MS methodology incorporating full-scan and multi-reaction monitoring acquisition mode, which is an effective tool for exploring intestinal microbial metabolism [37]. Furthermore, GC-MS finds extensive utility in plant metabolomics [38], microbiology [39], and clinical metabolomics [40].

### 2.3. Liquid Chromatography–Mass Spectrometry

LC-MS circumvents the complicated sample pretreatment in GC-MS, while ultra-performance liquid chromatography (UPLC) offers advantages such as high separation efficiency, rapid analysis speed, high detection sensitivity, and broad application scope. In comparison to GC, UPLC has been proven to be more suitable for the separation and analysis of compounds with elevated boiling points, macromolecules, and those with diminished thermal stability. LC-MS ionization sources include electrospray ionization (ESI), atmospheric pressure chemical ionization, atmospheric pressure photoionization, and fast atom bombardment. Presently, ESI represents a favored approach for LC-MS metabolomics investigations due to its “soft ionization” capability, which generates ions through charge exchange within the solution and typically results in intact molecular ions, aiding in initial recognition [29].

LC can be categorized into reverse-phase liquid chromatography (RPLC) and hydrophilic interaction liquid chromatography (HILIC). RPLC usually employs C18 columns to separate semi-polar compounds, including phenolic acids, flavonoids, alkaloids, and other glycosylated species. On the other hand, HILIC usually employs aminopropyl columns to separate polar compounds such as sugars, amino acids, carboxylic acids, and nucleotides [41]. Recently, two-dimensional and multidimensional liquid chromatography have gained prominence as potent platform technologies capable of enhancing peak capacity and resolution [42]. Nevertheless, there is currently no single chromatographic mode that can comprehensively analyze the entire metabolome with a single analysis. The integration of multiple analytical platforms facilitates improved metabolic coverage.

## 3. Metabolomics in Diabetic Retinopathy

**Figure 1:**
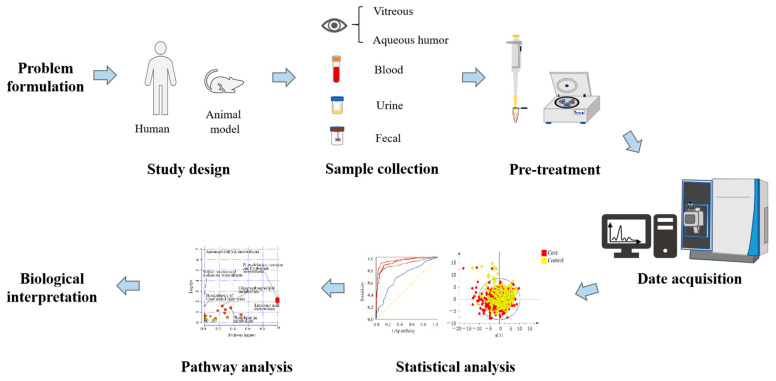
Schematic diagram of the research process design of metabolomics for DR.

Metabolomics, emerging as a prominent branch in the field of “omics” sciences subsequent to genomics, proteomics, and transcriptomics, amalgamates high-throughput analysis techniques with bioinformatics. It encompasses the quantitative and qualitative assessment of metabolites, which are important intermediates and final products of metabolism. The retina, an integral component of the central nervous system, exhibits a distinctive high metabolic activity akin to the brain, making it an immensely active tissue with substantial energy requirements [43]. Consequently, employing metabolomics in the context of DR can offer insights into the underlying mechanisms of the disease, facilitate diagnosis, and enable disease monitoring (Figure 1).

**Figure 2:**
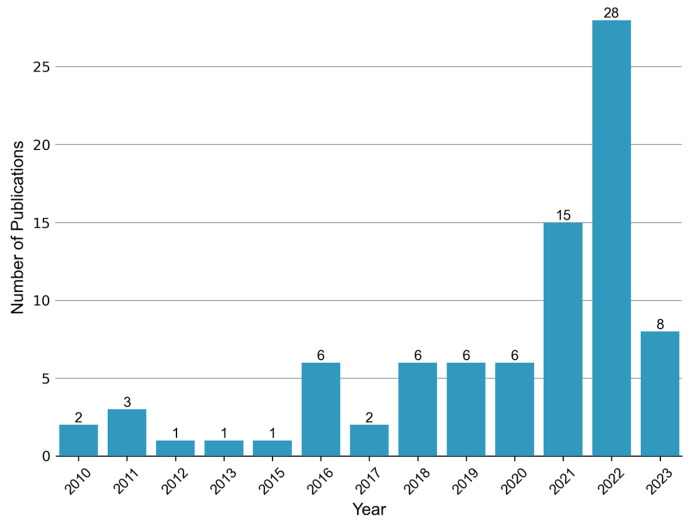
Annual publication trend of metabolomic studies in diabetic retinopathy. A total of 83 relevant articles were retrieved by searching the Web of Science and PubMed databases up until 5 June 2023. The search terms used were “diabetic retinopathy” AND (“metabolomics” or “metabonomics” or “metabolic profiling” or “metabolome”).

Ever since the publication of “Metabolic fingerprints of proliferative diabetic retinopathy: an ^1^H-NMR-based metabonomic approach using vitreous humor” in 2010 [44], there has been a growing body of research on the topic of metabolomics in the context of DR. This upsurge in studies, particularly observed in recent years (Figure 2), has predominantly focused on the analysis of vitreous and blood samples. However, several investigations have also explored alternative biological specimens including, but not limited to, aqueous humor, urine, and feces. Thus, in this review, we aim to summarize the key findings from metabolomics studies encompassing diverse sample matrices collected from individuals with DR.

### 3.1. Blood Metabolomics

Compared with other samples, a significant number of research studies have been undertaken in the field of blood-based metabolomics. This prevalence is primarily attributed to the relative ease of obtaining blood samples compared to other sample types. To further elucidate the subject, we compiled and summarized the key findings from previously published plasma metabolomics studies pertaining to DR in Table 1, as well as serum metabolomics studies in Table 2.

#### 3.1.1. Plasma Metabolomics

In the system of Western medicine, DR manifests in three intricate stages characterized by metabolic disorders: pre-clinical, NPDR, and PDR. In contrast, traditional Chinese medicine (TCM) categorizes DR into two syndrome types: non-Yang deficiency and Yang deficiency. The integration of TCM and Western medicine approaches has demonstrated promising results in alleviating fundus hemorrhage and diabetes-related symptoms. To discern and evaluate the similarities and differences between Western medicine staging and TCM syndrome-related biomarkers, plasma samples were collected and subjected to GC-TOFMS detection. Subsequent analysis identified that 10 metabolites exhibited the potential to discriminate between the various stages of Western medicine classification, while 4 metabolites could distinguish between the two TCM syndrome types. Notably, pyruvate and l-aspartic acid emerged as metabolites capable of differentiating both stages of DR according to Western medicine and TCM syndrome types. However, it is worth noting that the concentration of aspartic acid demonstrated an association with renal function, which is a potential confounding factor. In TCM theory, there is no significant correlation between aspartic acid concentration and renal function, and it is important to acknowledge the absence of pertinent renal-function-related information in this study [45]. Furthermore, another investigation indicated that the combination of glutamic acid and glutamine could improve the specificity of distinguishing DR from non-DR [48].

Furthermore, some studies have been conducted on the alterations in various types of amino acid metabolism through plasma metabolomics of DR patients. Sumarriva et al. identified 126 and 151 characteristic metabolites using LC-MS/MS to distinguish DR patients from diabetes, PDR, and NPDR patients, respectively. Among them, arginine, citrulline, dehydroxycarnitine, and glutamic c-semialdehyde can be used to effectively distinguish diabetes mellitus (DM) and DR patients, and carnitine served as a distinguishing factor between PDR and NPDR [49]. Arginine metabolic disorders may be related not only to urea cycle metabolites, but also to asymmetric dimethylarginine (ADMA) and nitric oxide. In order to gain further insight into their mechanistic roles, Peters et al. conducted a targeted metabolomics analysis on six arginine- and citrulline-related metabolites [53]. In comparison to the diabetic control group, plasma levels of arginine and citrulline were increased in DR patients, thus affirming the significance of arginine and citrulline metabolism in DR. Additionally, the study revealed elevated levels of plasma ADMA in PDR patients when compared to NPDR patients. However, this association lost significance after adjusting for creatinine values. Notably, a comparison between targeted and non-targeted metabolomics results unveiled the ability of citrulline and carnitine to differentiate the severity of DR using both approaches [55]. Combined with proteomics research, it has been discovered that tryptophan metabolism also plays a crucial role in the development of DR. Plasma tryptophan levels in PDR patients are lower than those in NPDR patients. The decrease in tryptophan will increase the content of vascular endothelial growth factor, which in turn promotes angiogenesis [52]. Moreover, using UHPLC-MS, Sun et al. identified 22 differentially expressed metabolites associated with different metabolic pathways and demonstrated the significance of 4 circulating plasma metabolites. Risk score analysis revealed a positive correlation between these metabolites and glycated hemoglobin levels [51]. Additionally, the combined use of alanine, histidine, leucine, pyruvate, tyrosine, and valine exhibited a superior correlation with type 2 diabetes and diabetic microangiopathy, which when combined may contribute to the subsequent triage of diabetic complications [67].

Numerous other types of metabolites have also been discovered as potential biomarkers for DR. Xia et al. employed a quantitative approach utilizing high-performance liquid chromatography coupled with ultraviolet and tandem MS to detect six pyrimidine-related metabolites. Significantly increased concentrations of cytosine, cytidine, and pyrimidine were observed in DR patients compared to the DM group. Receiver-operating characteristic (ROC) analysis revealed that cytidine exhibited superior performance as a potential marker, with an area under the curve (AUC) of 0.849 ± 0.048. The cytidine concentration of 0.076 mg/L was set as the cutoff point for distinguishing disease status, yielding a sensitivity of 73.7% and a specificity of 91.9% [46]. In another comparison between PDR and NPDR, cytidine displayed an AUC of 0.95. Moreover, fumaric acid, uridine, and acetic acid demonstrated potential as biomarkers for PDR, with AUCs of 0.96, 0.95, and 1.0, respectively. Notably, this study unveiled fumaric acid as a novel metabolite marker for DR, offering insights into potentially novel pathogenic pathways associated with DR [50]. Polyunsaturated fatty acids (PUFAs) and their metabolites have also demonstrated beneficial effects on various pathological processes, including diabetes. After screening and statistically analyzing the detected eicosane compounds, it was found that prostaglandin 2a (PGF2a) had a protective effect on NPDR patients. In vivo and in vitro experiments suggested that PGF2a may regulate the migration of retinal pericytes through FP receptors and reverse some retinal capillary damage, thus conferring a protective role [47]. In addition, the biosynthesis pathways of pantothenate and coenzyme A exhibited significant disruption and decreased plasma levels in DR patients, potentially attributed to impaired vitamin reabsorption in renal tubules, leading to reduced pantothenate conversion rates [54].

#### 3.1.2. Serum Metabolomics

Serum metabolomics studies on DR have also shed light on alterations in amino acid metabolism. The changes of tryptophan metabolism in the pathophysiology of diabetes and its related complications have been confirmed. However, in the serum metabolomics study of NPDR and PDR patients, tryptophan metabolite levels were found to increase, but tryptophan itself did not exhibit significant changes [56]. Another high-throughput targeted metabolomics study identified 16 metabolites as common specific metabolites of NPDR and PDR. Among them, total dimethylamine, tryptophan, and total tryptophan were identified as potential factors contributing to the progression of DR in DM patients. Variations in the findings related to tryptophan could be influenced by factors such as sample size and different disease groups. The results further support the exploration of tryptophan and kynurenine in the treatment and understanding of DR [59]. A case–control study employing propensity score matching identified a set of multidimensional network biomarkers containing linoleic acid, nicotinic acid, ornithine, and phenylacetylglutamine with high specificity and sensitivity (96% and 78%, respectively) in distinguishing DR from patients with type 2 diabetes mellitus (T2DM). This multidimensional network biomarkers system may present the most effective means for identifying DR [61]. New DR-related metabolic changes, such as thiamine metabolic disorders, reduced trehalose, and increased choline and indole derivatives, were also revealed in another similar case–control study [62]. In order to gain further insights into the metabolic changes from T2DM to DR, Wang et al. conducted comparative analyses of metabolic profiles across various stages, including NPDR and T2DM patients, PDR and T2DM patients, as well as DR and non-DR patients. The study was the first to confirm the close association of serum phosphatidylcholine and 13-hydroperoxyoctadeca-9,11-dienoic acid levels with different stages of DR in T2DM in Asian populations. Furthermore, abnormalities in other pathways, such as arginine biosynthetic metabolism, linoleic acid metabolism, aspartic acid, and glutamic acid metabolism, were observed in DR patients of Asian population [64].

With the advancement of multi-platform and multi-omics analyses, novel potential metabolic biomarkers for DR have been discovered. The integration of lipidomics and metabolomics can characterize subtle disturbances in response to lipid and metabolic changes and provide new insights into diseases. Curovic et al., combining metabolomics and lipidomics approaches, discovered four metabolites that were associated with different stages of DR, and three triglycerides exhibiting a negative correlation with the DR stage. Among them, 3,4 dihydroxybutyric acid was identified as an independent marker for DR progression [57]. Retinopathy in DR encompasses various manifestations, including microaneurysms, hard exudates (HEs), soft exudates, fibrous hyperplasia, and neovascularization [68]. Persistent HEs will develop into subretinal fibrosis with irreversible visual loss. By studying the lipidomics and metabolic profiles of patients with different severity levels of HEs, 19 metabolites and 13 HE-related pathways were identified. The combination model containing 20 lipids, such as triglyceride, ceramide, and N-acylethanolamine, demonstrated the most effective discrimination ability for HEs, with an area under the curve of 0.804 [66]. Utilizing GC-MS metabolomics, LC-MS metabolomics, and LC-MS lipidomics, comprehensive insights on the metabolic pathways involved in DR development have been obtained, leading to the identification and verification of a novel biomarker panel. The panel, consisting of 12-hydroxyeicosatetraenoic acid and 2-piperidone biomarkers, showed high sensitivity and specificity in distinguishing NPDR and NDR (0.929 and 0.901), which was higher than that of HbA1c (0.611 and 0.686), underscoring its potential for early diagnosis [58]. DR represents a form of diabetic microangiopathy characterized by endothelial dysfunction associated with microvascular complications. By combining metabolomics and proteomics analyses of serum exosomes, it was found that the up-regulation of an alpha subunit of the coagulation factor fibrinogen (FIBA) and the down-regulation of 1-methylhistidine contribute to diabetic endothelial dysfunction, impacting both macrovascular and microvascular complications. However, further cohort studies are required to elucidate the specific role of FIBA and 1-methylhistidine in the development of DN and DR [65].

In addition to the abovementioned MS-based serum metabolomics analysis, there are various NMR-based metabolomics studies and studies combining metabolomics and algorithms to develop an optimal model for DR prediction. In a cross-sectional study involving three cohorts from China, Malay, and India, 16 serum metabolites associated with DR were identified using NMR technology. Among these metabolites, three were found to be significantly correlated with each stage of DR. Notably, elevated levels of tyrosine and cholesteryl ester to total lipid ratio demonstrated a protective effect against severe DR, whereas increased levels of creatinine were positively associated with all three DR outcomes [60]. Furthermore, Li et al. employed metabolomics techniques and machine learning algorithms, based on propensity score matching approach, to establish a nomogram model for DR prediction. This model incorporated factors including diabetes duration, systolic blood pressure, and thiamine triphosphate. Impressively, the developed nomogram model exhibited excellent classification performance, with AUCs (95% CI) of 0.99 (0.97–1.00) and 0.99 (0.95–1.00) in both the training and testing sets, respectively [63]. These findings provide valuable insights into the management and control of DR, thereby contributing to the advancement of this field.

### 3.2. Vitreous Metabolomics

The vitreous body serves as a water medium in direct contact with the retina, lens, and numerous cells. It contains valuable information regarding the etiology of eye and vitreoretinal diseases [69]. We summarized the main findings from previous studies on the vitreous metabolome in individuals with DR (Table 3). Barba et al. utilized ^1^H-NMR to conduct a vitreous metabolomics analysis, aiming to explore the metabolic differences between macular hole surgery in non-diabetic patients and patients with type 1 diabetes accompanied by PDR. Partial least squares discriminant analysis was employed to develop a recognition model. The results demonstrated that, after removing the lactic acid peak, 19 out of 22 PDR patients and 18 out of 22 controls were accurately classified, yielding a sensitivity rate of 86% and a specificity rate of 81% [44]. Notably, the vitreous samples of PDR patients exhibited a significant depletion of ascorbic acid and galactose, along with elevated levels of lactic acid. However, the authors emphasized the study’s limitations, considering that vitreous hemorrhage frequently occurs in PDR patients, which may render these identified metabolites unrelated to DR. In a separate investigation by Wang et al., a comparison between plasma and vitreous metabolic profiles was conducted. Interestingly, five metabolites were found to be overlapping. Specifically, phenylacetylglutamine exhibited a significant increase, whereas valeric acid displayed a significant decrease, contrary to previous studies. The authors hypothesized that these discrepancies may be attributed to racial variations [54].

Using UHPLC-MS non-targeted metabolomics analysis, it has been determined that purine metabolite xanthine serves as the primary biomarker for distinguishing individuals with DR from healthy controls. Moreover, proline and citrulline play essential roles in differentiating DR from control subjects well as those with rhegmatogenous retinal detachment (RD). Within the vitreous of individuals with DR, downstream glycolysis metabolites, such as glyceraldehyde 3-phosphate and 2/3-phosphoglycerate, as well as the ratio of lactic acid to pyruvic acid, demonstrate a significant decrease [70]. However, when comparing DR patients with non-diabetic patients with macular hole (MH), vitreous lactic acid levels are significantly higher in those with PDR [44]. Another study, utilizing GC-TOFMS, identified a group of metabolites (d-2,3-dihydroxypropanoic acid, isocitric acid, threonic acid, pyruvic acid, l-Lactic acid, pyroglutamic acid, fructose 6-phosphate, ornithine, l-threonine, l-glutamine, and l-alanine) that exhibited good discriminatory potential between PDR and control subjects [71]. More recently, untargeted UHPLC-MS/MS analysis identified creatine as a potential target for PDR, and the supplementation of creatine in mouse models demonstrated inhibitory effects on pathological retinal neovascularization [72].

It is worth noting that the biochemical changes observed in the vitreous closely mirror alterations in retinal homeostasis. However, obtaining vitreous samples from healthy controls is considerably challenging, as it necessitates invasive surgical procedures. This limitation poses a hindrance to the advancement of vitreous metabolomics research.

### 3.3. Aqueous Humor Metabolomics

In the preclinical stage of DR, vitrectomy is not applicable, and obtaining vitreous samples for analysis is not feasible due to the inapplicability of vitrectomy. As an alternative, aqueous humor is a transparent liquid synthesized by ciliary epithelial cells. It circulates through the anterior and posterior chambers of the eye and eventually drains into the veins, providing nourishment to avascular ocular tissues and facilitating the removal of metabolic waste [73]. Aqueous humor has emerged as a viable substitute for vitreous samples in metabolomics investigations related to DR.

Studies have sought to explore the correlation between metabolite levels in aqueous humor and vitreous samples from individuals with DM and DR. Specifically, oxidized glutathione trisulfide, cystine, and cysteine persulfide levels were found to be correlated across aqueous and vitreous samples [74]. Moreover, a comparative analysis of metabolites in aqueous humor and vitreous revealed the presence of eight metabolites in aqueous humor samples, with three of these metabolites (citrulline, inositol, and d-glucose) also observed in vitreous samples [71]. These findings clearly illustrate the potential utility of aqueous humor as a suitable substitute for vitreous samples in metabolomics analyses pertaining to DR. Consequently, the use of aqueous humor in lieu of vitreous samples circumvents the challenges associated with obtaining vitreous samples in the preclinical stage of DR. This enables the investigation of metabolic alterations and biomarkers that help us understand the pathology of DR and may have implications in early diagnosis, therapeutic interventions, and improved clinical management strategies for this condition.

Limited research has focused on metabolomics studies involving human aqueous humor samples in the context of DR. One investigation utilized ^1^H-NMR, employing 2D homonuclear TOCSY and 2D pulsed-field gradient COSY, to compare and analyze aqueous humor samples derived from patients with DM and cataracts, as well as DR and cataracts, alongside elderly individuals with cataracts. Notably, this study represents the first comprehensive metabolomics analysis study based on ^1^H-NMR to explore the differential metabolic spectrum of aqueous humor in patients with DR. Following a comprehensive series of analyses, the study revealed several metabolites exhibiting the highest degree of variability. Notably, succinic acid, lactic acid, asparagine, histidine, glutamine, and threonine were identified as the most variable metabolites [75]. The identification of these distinct metabolic signatures within aqueous humor samples from DR patients adds valuable insights into the metabolic alterations associated with the disease. Nonetheless, additional studies involving larger sample sizes and rigorous validation are required to substantiate these findings and further enhance our understanding of the underlying metabolic perturbations in DR. These findings highlight the potential utility of aqueous humor metabolomics as a valuable approach to investigate the metabolic changes underlying DR. Moreover, this research may facilitate the identification of relevant biomarkers and inform the development of targeted therapeutic interventions. Continued investigations in this area hold significant promise for advancing our knowledge of DR pathogenesis and enhancing clinical management strategies for this sight-threatening condition.

### 3.4. Urine Metabolomics

Urine, being easily collectible and rich in metabolites, has emerged as a valuable source for non-invasive biomarker discovery in metabolomics studies [76]. Significantly, the field of urine metabolomics has gained prominence for its ability to reflect the imbalance of biochemical pathways in vivo.

In a study based on NMR technology used for the quantitative analysis of urine samples from two cohort populations in China and India, metabolites were examined for their correlation with different stages of DR, including any DR, moderate/severe DR, and vision-threatening DR. The analysis revealed that 10 metabolites (citrate, ethanolamine, formate, hypoxanthine, 3-hydroxyisovalerate, 3-hydroxyisobutyrate, alanine, glutamine, uracil, and glycolic acid) were associated with at least one of the DR stages. Among them, citrate, ethanolamine, formate, and hypoxanthine displayed a negative correlation with all three DR results [60]. In another investigation conducted by Wang et al., UPLC-MS was employed to study the metabolomics of DR in rat urine upon the administration of Bushen Huoxue prescription. In this study, nine potential biomarkers were identified, including cholic acid, *p*-cresol sulfuric acid, 5-l-glutamyl taurine, 3-methyldiglucoside, nephropathy and phenylacetylglycine, 3-methyldioxyindole, kynurenic acid, hippuric acid, indoxyl sulfate, cholic acid, *p*-cresol sulfate, *p*-cresol glucuronide, and 5-L-glutamyl-taurine. These biomarkers were found to be significantly correlated with tryptophan metabolism, lipid metabolism, and intestinal microbial metabolism [77]. Moreover, the urine metabolomics analysis of diabetic model rats demonstrated the impact of exogenous free N^ε^-(carboxymethyl) lysine intake on various metabolic pathways, such as amino acid metabolism, TCA cycle, and carbohydrate metabolism [78]. In summary, urine metabolomics presents a promising avenue for non-invasive biomarker discovery in the context of DR. These studies demonstrate the potential of metabolite profiling in urine to identify specific biomarkers associated with different stages of DR and uncover metabolic perturbations. Further research in this field will contribute to an enhanced understanding, diagnosis, and management of DR.

### 3.5. Fecal Metabolomics

Recent studies have highlighted the significant influence of gut microbiota in the pathogenesis of diabetic complications, including the development of DR [79,80]. Fecal metabolomics provides a valuable approach for examining the metabolic interactions between the host, diet, and gut microbiota, enabling an in-depth exploration of their role in disease processes [81]. Moreover, emerging studies have begun to elucidate the connection between gut microbiota and ocular abnormalities, including uveitis, glaucoma, and age-related macular degeneration, leading to the proposition of the microbiota–gut–retina axis concept [80].

To explore the relationship between gut microbial metabolism and DR, fecal samples were subjected to metabolomic analysis using UHPLC-MS and LC-MS. In a study by Li et al., non-targeted metabolomic analysis was performed on stool samples obtained from DR and non-DR in type 2 diabetic patients. The findings revealed significant increases in *Acidaminococcus*, *Escherichia coli*, and *Enterobacteriaceae* in patients with DR, while bifidobacteria and lactic acid bacteria exhibited a significant decrease. Additionally, the proportion of Pasteurella was significantly reduced [82]. Moreover, the bacterial abundance and diversity of intestinal flora were found to be significantly lower in diabetic patients with PDR compared to those without DR. Furthermore, fecal metabolomics analysis demonstrated an elevation in arachidonic acid metabolites, including hydroxyeicosatetraenoic acid and leukotrienes, which are known mediators in the development of DR, in PDR patients [83]. In a comparison between DR patients and healthy individuals, DR patients exhibited an enrichment of fecal bacteria belonging to the Rochalimaea and Longevia genera, while Akermann bacteria are reduced. Furthermore, carnosine, succinic acid, niacin, and niacinamide levels in the DR group were significantly lower than those in the healthy control group. The KEGG annotation of metabolomic data revealed 17 pathways with substantial differences in metabolite composition between DR patients and healthy controls, while only 2 pathways exhibited significant differences between DR patients and DM patients [84].

### 3.6. Other Biological Samples: Metabolomics

In addition to the aforementioned types of samples, metabolomics studies on DR have also been conducted utilizing cerebrospinal fluid (CSF) and retinal samples. CSF, although a rare sample in DR metabolomics research, has been employed in certain investigations. For instance, the combination of alanine, histidine, leucine, pyruvate, tyrosine, and valine in CSF exhibited strong correlations with the presence of T2DM (AUC:0.951) and DR (AUC:0.858) [67]. Moreover, Wang et al. employed non-targeted UPLC-MS/MS and GC-MS techniques to explore the metabolic characteristics of retinas in streptozotocin-induced diabetic mice. Pathway enrichment analysis revealed alterations in 50 metabolic pathways. In conjunction with transcriptomics analysis, these findings suggest potential disturbances in the Wahlberg effect, amino acid metabolism, and retinol metabolism, shedding light on potential metabolic mechanisms and therapeutic targets for DR [85].

## 4. Metabolomics Studies in DR Models

At present, the main animal models used in DR research can be divided into two primary categories: induced animal models and genetic animal models [86,87], as summarized in Table 4. However, only a limited number of models have been utilized for metabolomics studies.

Among these models, the streptozotocin (STZ)-induced diabetic rat or mouse model has gained widespread use due to its similarity to the pathological changes observed in early DR [88]. Thus, this model has become the most commonly employed in DR research. Notably, a study investigating retinal metabolomics in STZ-induced diabetic mice revealed that the Warburg effect may play a pivotal role in the pathogenesis of DR. In the retina of diabetic mice, ornithine was significantly increased, proline was decreased, and arginine metabolism was changed, which were similar to the metabolic changes in the plasma and vitreous of DR patients [85]. The consistency between mouse retina and human samples indicates that STZ-induced diabetic mice can be used as valuable tools for the further exploration of metabolic mechanisms underlying DR. Additionally, comprehensive metabolomics, lipidomics, and RNA profiling studies conducted on the retinas of STZ-induced diabetic mice uncovered that activated microglia exhibit distinct metabolic characteristics and serve as the primary source of pro-inflammatory cytokines in early DR. Furthermore, the intracellular metabolic microenvironment, particularly glycolysis, appears to reprogram retinal inflammation. These findings pave the way for potential future studies aiming to reprogram the intracellular metabolism of retinal-specific microglia to mitigate local inflammation and prevent the progression of early DR [89].

**Table 4:** Animal models for studying DR.

Category		Animal Models
Induced model		STZ-induced models
		Alloxan-induced models [90]
		Diet-induced models
		Oxygen-induced models
Genetic models	Mice model	Ins2Akita [91]
		Non-obese diabetic [92]
		Leprdb [93]
		Kimba [94]
		Akimba [95]
	Rat model	Zucker diabetic fatty [96]
		Otsuka Long-Evans Tokushima Fatty [97]
		Biobreeding diabetes-prone [98]
		Wistar Bonn Kobori [99]
		Goto–Kakizaki [100]
		Spontaneously diabetic Torii [96]

However, it is important to note that STZ-induced models are unlikely to exhibit retinal neovascularization, which is a key pathological feature of PDR. Thus, researchers have turned to the oxygen-induced mouse retinopathy (OIR) model, which closely mimics the pathological manifestations observed in PDR patients, for investigating PDR [101]. In the vitreous of PDR patients, alterations in creatine and creatine-related pathways have been observed. Decreased levels of creatine have been found in the vitreous of PDR patients, as well as in the retina of the OIR model [72]. Moreover, creatine supplementation has been shown to reduce retinal neovascularization in OIR, suggesting its potential as a therapeutic target for PDR. However, it is important to recognize that while the OIR model simulates neovascularization in the retina, it does not encompass other aspects of diabetes that occur prior to neovascularization [72]. Consequently, further investigations are required to fully elucidate the underlying mechanism of creatine and its potential implications in DR.

The variations in metabolic changes observed among different induction or genetic models underscore the multifactorial nature of DR. In the future, animal-model-based metabolic studies will help to improve our understanding of the pathogenesis and progression of DR.

## 5. Dysregulation of Metabolic Pathways in DR

DR is a complex and chronic metabolic disease marked by the dysregulation of multiple metabolic pathways. Metabolomics studies have revealed that several metabolic pathways are disrupted in DR, including those associated with amino acid metabolism, energy metabolism (TCA cycle, glycolysis, and carnitine metabolism), pyrimidine metabolism, and lipid metabolism [49,58,71,102].

The retina, as a highly metabolically active tissue, has a significant energy demand. Glycolysis and the TCA cycle play crucial roles in DR. Glycolysis converts glucose into pyruvic acid, which can undergo anaerobic metabolism, leading to the production of lactic acid [67]. Moreover, pyruvic acid can be oxidized to generate acetyl-CoA, fueling the TCA cycle. Within the TCA cycle, succinic acid is an intermediate metabolite that plays a pivotal role in adenosine triphosphate production within the mitochondria. Therefore, succinic acid may hold potential as a biomarker for DR. Studies by Jin et al. demonstrated significant reductions in lactic acid and succinic acid levels in the aqueous humor of DR patients. However, in the vitreous humor, succinic acid levels were significantly increased and succinic acid may accumulate in the retina with an insufficient oxygen supply [75].

In the context of amino acid metabolism, the arginine metabolic pathway takes prominence and has been investigated in aqueous humor, plasma, serum, and vitreous metabolomics studies. Arginine can be metabolized through two different pathways: the arginase pathway, which involves the enzyme arginase II (Arg-II) and leads to the production of ornithine and urea, and the nitric oxide synthase (NOS) pathway, which produces citrulline and nitric oxide (NO) [64]. Ornithine has been linked to chronic inflammatory injury mediated by microglia and macrophages observed in type 2 diabetes. Elevated ornithine levels indicate an increase in Arg-II enzyme activity, potentially resulting in reduced NOS pathway activity, which predominantly synthesizes NO. NO is a crucial vasodilator that plays a vital role in maintaining vascular endothelial health [49]. Insufficient NO levels, combined with elevated levels of polyamines and proline, can lead to endothelial cell dysfunction and impaired vasodilation, and can also stimulate cell proliferation and fibrosis [55]. However, it should be noted that while arginine can stimulate insulin release in pancreatic β cells, poorly regulated arginine and citrulline levels can also contribute to retinal endothelial cell dysfunction [55,103]. Moreover, the metabolism of tryptophan, glutamic acid, and alanine has also been associated with the development of DR, although their precise mechanisms remain unclear [63,85].

Pyrimidine, a crucial component of DNA and RNA, performs various essential biological functions. Recent studies have revealed the association between pyrimidine metabolism and DR [46]. Pyrimidines participate in cellular processes as building blocks of genetic material and have implications for diverse cellular activities. Sphingolipids, on the other hand, have emerged as important components of lipids with pivotal roles in signal transduction, cell proliferation, apoptosis, and membrane structure. In recent years, their involvement in various cellular processes has been recognized. The sphingosine kinase 1/sphingosine 1 phosphate pathway, in particular, has been associated with cell fibrosis through its ability to stimulate mesangial cell proliferation and matrix formation. The plasma levels of sphingosine 1 phosphate were observed to be higher than those in individuals with DR compared to healthy individuals, and these levels demonstrated a positive correlation with HbA1c levels [102].

## 6. Conclusions and Future Perspectives

Metabolites are the final products of various changes in genome, transcriptome and proteome. Metabolomics offers a powerful approach to unravel the mechanisms underlying metabolic disorders and diseases, including DR. Metabolomics studies have provided insights into the metabolite changes that occur in various biological samples and have identified numerous potential biomarkers and therapeutic targets for DR. However, metabolomics is still in the early stage of development, and there are still many problems to be solved.

Firstly, the metabolomics database is still limited, and current findings may only represent a fraction of the overall etiology of DR. Moreover, differences in demographic data, such as race, region, and age, can affect outcomes and mask the direct effects of disease. Therefore, standardization of data analysis and reporting between institutions is essential for absolute quantification and reliable statistical results. Large-scale clinical metabolomics studies using standardized protocols and validated findings are necessary to reduce individual differences and improve the reliability of results. Additionally, advancements in metabolomics technology and analysis platforms are crucial for expanding metabolic coverage. Techniques such as hyperpolarized nuclear magnetic resonance and cryogenic-probe-based Rheo-NMR enhance spectral resolution and metabolite identification. Two-dimensional and multidimensional liquid chromatography techniques can offer enhanced peak capacity and improved resolution. The combination of multiple analysis platforms can help improve metabolic coverage, and simultaneous utilization of complementary analytical platforms can facilitate a more comprehensive understanding of the underlying biological processes.

Moreover, the integration of metabolomics with other omics approaches, such as transcriptomics and proteomics, holds great potential for unraveling the complex mechanisms driving the occurrence and progression of DR. This comprehensive approach can help to discover new biomarkers and effective therapeutic targets. In future studies, it is important to prioritize the development of non-invasive, rapid, and cost-effective DR biomarkers, which will greatly enhance our understanding of the complex pathogenesis of the disease.

In summary, metabolomics has demonstrated its power as a tool for characterizing metabolic alterations in DR and offers great potential for providing valuable insights into the disease. Standardization, large-scale studies, technological advancements, and interdisciplinary collaborations are key to advancing our understanding of DR and improving its clinical management.

## Figures and Tables

**Table 1:** Plasma metabolomics of DR clinical populations.

References	Subjects	Techniques	Statistical Methods	Differential Metabolites
Li X (2011) [45]	NPDR (n = 39) PDR (n = 25) DM (n = 25) Control (n = 30)	GC-TOFMS	PLS-DA	Pyruvic acids, L-aspartic acid, β-hydroxybutyric acid, methymaleic acid, citric acid, glucose, stearic acid, transoleic acid, linoleic acid, and arachidonic acid
Xia JF (2011) [46]	DR (n = 38) DM (n = 37) Control (n = 41)	HPLC–UV/MS/MS	ROC	Cytosine, cytidine, uridine, thymine, thymidin, and 2′-deoxyuridine
Peng LY (2018) [47]	NPDR (n = 28) Control (n = 22)	LC-MS	OPLS-DA	Prostaglandin 2a
Rhee (2018) [48]	NPDR (n = 72) PDR (n = 52) Control (n = 59)	GC-TOF-MS, UPLC-Q-TOF-MS	OPLS-DA	Asparagine, aspartic acid, glutamine, glutamic acid, 1,5-anhydroglucitol, fructose, and *myo*-inositol
Sumarriva (2019) [49]	NPDR (n = 49) PDR (n = 34) Control (n = 90)	LC-MS/MS	PLS-DA	Arginine, citrulline, glutamic c-semialdehyde, acylcarnitine, and dehydroxycarnitine
Zhu XR (2019) [50]	PDR (n = 21) NDR (n = 21)	LC-MS	ROC	Fumaric acid, uridine, acetic acid, and cytidine
Sun Y (2021) [51]	DR (n = 42) Control (n = 32)	UHPLC-QE MS	OPLS-DA	Pseudouridine, N-acetyltryptophan, glutamate, leucylleucine, and HbA1c
Ding C (2022) [52]	PDR (n = 27) NPDR (n = 18) Control (n = 21)	UPLC-MS	OPLS-DA	Proline, threonine, glutamine, aspartate, glutamate, and tryptophan
Peters (2022) [53]	DM (n = 159) NPDR (n = 92) DR (n = 64)	LC-MS/MS	Wilcoxon Rank Sum test	Arginine, citrulline, asymmetric dimethylarginine, ornithine, proline, and argininosuccinic acid
Wang HY (2022) [54]	PDR (n = 88) Control (n = 51)	UPLC-MS/MS	OPLS-DA	Phenylacetyl glutamine, pantothenate, CoA, tyrosine, and phenylalanine
Wang ZY (2022) [55]	NPDR (n = 28) PDR (n = 28) DM (n = 27) Control (n = 27)	UHPLC-MS/MS	OPLS-DA	L-Citrulline, indoleacetic acid, 1-methylhistidine, phosphatidylcholines, hexanoylcarnitine, chenodeoxycholic acid, and eicosapentaenoic acid

**Table 2:** Serum metabolomics of DR clinical populations.

References	Subjects	Techniques	Statistical Methods	Differential Metabolites
Munipally (2011) [56]	NPDR (n = 22) PDR (n = 24) Control (n = 35)	HPLC	*t*-test	kynurenine, kynurenic acid, and 3-hydroxykynurenine
Curovic (2020) [57]	Mild PDR (n = 90) Moderate PDR (n = 186) PDR (n = 121) PDR with fibrosis (n = 107) Control (n = 141)	GC-TOFMS	Cox models	2,4-dihydroxybutyric acid, 3,4-dihydroxybutyric acid, ribonic acid, and ribitol
Xuan QH (2020) [58]	NDR (n = 111) NPDR (n = 99) MMPDR (n = 90) SNPDR (n = 85) PDR (n = 76)	GC-MS, LC-MS	PLS-DA	12-hydroxyeicosatetraenoic acid and 2-piperidone
Yun JH (2020) [59]	NDR (n = 143) NPDR (n = 123) PDR (n = 51)	LC-MS/MS	Stats	Total dimethylamine, tryptophan, kynurenine, carnitines, several amino acids, and phosphatidylcholines
Quek (2021) [60]	Moderate/above DR (n = 328) VTDR (n = 217) Control (n = 2211)	NMR	ROC	Tyrosine, 3-hydroxybutate, sphingomyelins, and creatinine
Zuo JJ (2021) [61]	DM (n = 46) DR (n = 46)	UPLC-ESI-MS/MS	OPLS-DA	Linoleic acid, nicotinuric acid, ornithine, and phenylacetylglutamine
Guo CG (2022) [62]	NPDR (n = 60) PDR (n = 9)	UPLC-MS/MS	PLS-DA	12-/15-HETE, PUFAs, thiamine triphosphate, L-cysteine, and glutamate
Li JS (2022) [63]	NDR (n = 112) DR (n = 83) Control (n = 755)	UPLC-ESI-MS/MS	ROC	Thiamine triphosphate and 2-pyrrolidone
Wang ZY (2022) [64]	NPDR (n = 15) PDR (n = 15) DM (n = 15) Control (n = 15)	UHPLC-MS/MS	PLS-DA	Aspartate, glutamine, N-acetyl-L-glutamate,N-acetyl-L-aspartate, pantothenate, dihomo-gamma-linolenate, docosahexaenoic acid, and icosapentaenoic acid
Yang J (2022) [65]	DR + DN (n = 20) Control (n = 20)	UPLC-MS/MS	OPLS-DA, PLS-DA	1-methylhistidine, coagulation factor, and fifibrinogen
Shen YH (2023) [66]	NPDR (n = 105) PDR (n = 62)	LC-MS	ROC, PLS-DA	Methionine and taurine

**Table 3:** Vitreous humor metabolomics of DR clinical populations.

References	Subjects	Techniques	Statistical Methods	Differential Metabolites
Barba (2010) [44]	PDR (n = 22) Controls (n = 22)	^1^H-NMR	PLS-DA	Lactate, acetate, galactitol, ascorbic acid, and ribose phosphate
Nathan R (2018) [70]	DR (n = 8) RD (n = 17) Controls (n = 9)	UHPLC-MS	PLS-DA, ROC	Xanthine, proline, citrulline, and long-chain acylcarnitines
Wang HY (2020) [71]	PDR (n = 28) MH (n = 22)	GC-TOFMS	OPLS-DA, ROC	Pyruvic acid, uric acid, ornithine, l-lysine, l-leucine, pyroglutamic acid, l-alanine, l-threonine, hydroxylamine, l-valine, l-alloisoleucine, l-phenylalanine, creatinine, myoinositol, and l-glutamine
Tomita (2021) [72]	PDR (n = 35) Control (n = 19)	UHPLC-MS/MS	*t* test	Pyruvate, lactate, proline, glycine, citrulline, ornithine, allantoin, creatine, dimethylglycine, *N*-acetylserine, succinate, and α-ketoglutarate
Wang HY (2022) [54]	PDR (n = 51) Control (n = 23)	UPLC-MS/MS	OPLS-DA	Phenylacetyl glutamine, pantothenate, CoA, tyrosine, and phenylalanine
